# An insight into the different responses to salt stress in growth characteristics of two legume species during seedling growth

**DOI:** 10.3389/fpls.2023.1342219

**Published:** 2024-01-24

**Authors:** Jia Mi, Xinyue Ren, Jing Shi, Fei Wang, Qianju Wang, Haiyan Pang, Lifang Kang, Changhui Wang

**Affiliations:** ^1^Shanxi Key Laboratory of Ecological Restoration on Loess Plateau, Institute of Loess Plateau, Shanxi University, Taiyuan, China; ^2^Field Scientific Observation and Research Station of the Ministry of Education for Subalpine Grassland Ecosystem in Shanxi, Ningwu, China; ^3^Shanxi Key Laboratory of Grassland Ecological Protection and Native Grass Germplasm Innovation, Shanxi Agricultural University, Taigu, China; ^4^College of Environment and Resources Sciences, Shanxi University, Taiyuan, China; ^5^Key Laboratory of Plant Resources and Beijing Botanical Garden, Institute of Botany, Chinese Academy of Sciences, Beijing, China; ^6^College of Grassland Science, Shanxi Agricultural University, Taigu, China; ^7^Observation and Research Station for Grassland Ecosystem in the Loess Plateau, Shanxi Agricultural University, Taigu, China

**Keywords:** salt stress, legume, seedling, morphological and physiological characteristics, protective enzyme system, structural equation modeling

## Abstract

Legumes play a crucial role in the restoration and utilization of salinized grassland. To explore the physiological response mechanism of *Astragalus membranaceus* and *Medicago sativa* seedlings to salt stress, salt stress culture experiments with five NaCl concentration treatments (0 mmol/L, 50 mmol/L, 100 mmol/L, 200 mmol/L, and 300 mmol/L) were conducted on these two legume seedlings. Morphological characteristics, physiological features, biomass, and the protective enzyme system were measured for both seedlings. Correlation analysis, principal component analysis (PCA), and membership function analysis (MFA) were conducted for each index. Structural equation modeling (SEM) was employed to analyze the salt stress pathways of plants. The results indicated that number of primary branches (PBN), ascorbate peroxidase (APX) activity in stems and leaves, catalase (CAT) activity in roots, etc. were identified as the primary indicators for evaluating the salt tolerance of *A. membranaceus* during its seedling growth period. And CAT and peroxidase (POD) activity in roots, POD and superoxide dismutase (SOD) activity in stems and leaves, etc. were identified as the primary indicators for evaluating the salt tolerance of *M. sativa* during its growth period. Plant morphological characteristics, physiological indexes, and underground biomass (UGB) were directly affected by salinity, while physiological indexes indirectly affected the degree of leaf succulence (LSD). Regarding the response of the protective enzyme system to salt stress, the activity of POD and APX increased in *A. membranaceus*, while the activity of CAT increased in *M. sativa*. Our findings suggest that salt stress directly affects the growth strategies of legumes. Furthermore, the response of the protective enzyme system and potential cell membrane damage to salinity were very different in the two legumes.

## 1 Introduction

In the context of global climate change, the threat of soil salinization is escalating. Globally, approximately 1.1 billion hectares of salt-affected land are considered unsuitable for growing crops, constituting 7% of the world’s land surface (Wicke et al., 2011). Soil salinization is a primary contributor to the shortage of land resources and the deterioration of the ecological environment. Therefore, it is necessary to utilize saline-alkali land to enhance agricultural production. In China, the area affected by saline-alkali soil is approximately 36 million hectares (Wang et al., 2021). Soil salinization is a critical adverse environmental factor that adversely affects seed germination, plant growth, and productivity, causing significant harm to the biosphere and ecological structure (Li and Li, 2022). Salt-tolerant plants play an active role in utilizing salinized soil, and the study of plant stress resistance under salt stress has become a focal point for botanists (van Zelm et al., 2020). Currently, the saline soil area in China is continuing to increase, and the cultivation of salt-tolerant crops, along with the development and utilization of salt-tolerant plant resources, represents feasible strategies to resist salt stress (Liu and Wang, 2021). Planting salt-tolerant crops can help address the inevitable global shortage of freshwater resources and the threat of soil salinization (Zhang et al., 2021).

Understanding the adaptive mechanisms of plants to saline–alkali stress and investigating the physiological and biochemical responses of plants under such stress are crucial endeavors for researchers. This exploration is essential for comprehending the intricate mechanisms of saline–alkali stress and enhancing the saline–alkali tolerance of plants (Fang et al., 2021). Salt stress exerts various influences on plants. For instance, plants subjected to salt stress undergo a series of physiological and biochemical changes aimed at regulating ion and water balance, thereby sustaining normal photosynthesis (Muchate et al., 2016). The effect of salt stress extends to seed germination, growth, photosynthetic pigments, photosynthesis, ion and nutrient balance, as well as overall productivity (Farooq et al., 2017; Zhou et al., 2023). In response to environmental challenges, plants activate regulatory mechanisms to mitigate salt-induced damage. Osmotic regulation, activation of antioxidant enzymes, and application of exogenous substances represent effective strategies employed by plants to alleviate salt stress (Feng et al., 2023).

Salt stress is a prominent abiotic factor significantly affecting plant growth, development, and yield. In saline-alkali soil, the symbiotic relationship between leguminous herbs and rhizobia not only facilitates salt reduction but also substantially enhances soil fertility during saline–alkali soil amelioration. Research on broad common beans subjected to salt stress revealed that elevated NaCl levels led to a reduction in plant height (PH), leaf area, and leaf number (Torche et al., 2018). Similarly, a 7-day salt stress treatment on soybeans demonstrated that NaCl inhibited overall plant growth (Ning et al., 2018). Studies in peas indicated that salt stress influenced sodium distribution in roots and buds, thereby inhibiting seedling growth and development (Tokarz et al., 2020). Most legumes are sensitive to high salt levels in the soil, and the  oil salt content affects almost all parameters of plant development (Shrivastava and Kumar, 2015). Investigations on mung beans underscored the inhibitory effect of salt stress on plant growth (Lim et al., 2022). Consequently, there is a need to intensify research on the growth characteristics of leguminous herbs under salt stress to establish a scientific foundation for assessing plant salt tolerance and understanding the underlying mechanisms.

While existing studies contribute significantly to unraveling the effect of salt on plants, the diverse evaluation indices for legume salt tolerance introduce complexity and hinder the establishment of a standardized index system. Furthermore, the underlying mechanisms driving legume characteristics’ response to salt stress remain unclear. Our research aims to address these gaps by exploring a method to screen the salt tolerance index system of legumes. And the response of legume varieties to salt stress along with the associated mechanisms of salt tolerance will be elucidated. We focused on two legume species, *Astragalus membranaceus* and *Medicago sativa*, conducting seedling growth experiments with varying NaCl concentrations. *A. membranaceus* is a medicinal plant with salt-resistance potential and economic value, and *M. sativa* is a high-yield forage plant with high salt tolerance. The objective was to observe changes in the physiological and biochemical characteristics of legume seedlings under NaCl stress, thereby comprehending the response process of the two legume types to salt stress and revealing the salt tolerance mechanism underpinning legumes’ response to salt stress.

## 2 Materials and methods

### 2.1 Legume plant species

Two legume species, namely *A. membranaceus* and *M. sativa*, were subjects of experimentation.

### 2.2 Experimental methods and procedures

#### 2.2.1 Cultivation of seedlings

Plant seedlings were grown in a well-ventilated plant culture laboratory at a temperature of 25 ( ± 1)°C and a humidity of 40%. The culture substrate of soil consisted of 20% vermiculite, 20% perlite, 10% humus, and 50% sand. The pots with a height of 85 mm and a diameter of 100 mm used in this experiment, in each pot, approximately 80–100 seeds were added. Six pots of each of the five gradients of the two plants were set up for seedling establishment. During the initial 30 days of plant growth, the Hoagland nutrient solution was applied every 5 days. Full-spectrum LED plant growth lamps were used to provide light. The light intensity was 3200 lx for 12 hours of light and 12 hours of dark per day. Upon reaching a seedling height of 8–10 cm, thinning was performed, ten plant individuals of similar height, leaf size, and leaf number were retained in each pot. One week post-thinning, the salt stress experiment commenced.

#### 2.2.2 Salt stress treatment

The experiment involved five levels of salt concentration treatment, and each treatment was replicated three times. Three plant individuals were selected as replicates for the determination of growth and physiological parameters. The designated salt treatment levels were as follows: 0 mmol/L (control check), 50 mmol/L, 100 mmol/L, 200 mmol/L, and 300 mmol/L. We set salt concentrations based on previous studies (Campanelli et al., 2013; Tani et al., 2018). To eliminate the interference of micro-environmental variations from the experimental results, the positioning of the cultivation basin was randomly changed frequently. Empirical observations were conducted over a 14-day period.

### 2.3 Parameter measurement

#### 2.3.1 Determination of seedling growth index

On the 13th day of treatment, the number of primary branches (PBN) of the main stem and the PH of each potted plant were recorded. Harvesting occurred on the 14th day, with measurements taken for leaf area (LA), the weight of fresh stem and leaf. Roots were washed and weighed fresh after removing sediment and excess water with absorbent paper. Stems, leaves, and roots were dried at 65°C until a constant dry weight was achieved. The root-to-shoot ratio (R/S) and the degree of leaf succulence (LSD) were calculated, representing the ratio of the dry weight of the root to the dry weight of the stem and leaf, and the ratio of fresh weight to the dry weight of leaves, respectively.

#### 2.3.2 Determination of seedling physiological indexes

Malondialdehyde (MDA) levels in plant seedlings were determined using thiobarbiturate oxidation colorimetry, and the activity of superoxide dismutase (SOD) was determined using the WST-8 method. The activity of peroxidase (POD), ascorbate peroxidase (APX), and catalase (CAT) was determined using guaiacol colorimetry, AsA colorimetry, and ammonium molybdate colorimetry, respectively.

### 2.4 Statistical analysis

Microsoft Excel 2016 and SPSS 24.0 were utilized for statistical data analysis. A correlation model was established, and Origin 2022 was employed for creating figures. Principal component analysis (PCA) was used to obtain the magnitude of each indicator’s contribution and eigenvectors, and the data to compare the magnitude of each indicator’s influence on plant salt tolerance. The salt tolerance of the two legumes was comprehensively evaluated by membership function analysis (MFA).

The relative biomass was calculated using the following formula:


(1) $$\text{Relative }\mathrm{biomass}=\frac{SB-CB}{CB}\times 100\%$$

where *SB* is the biomass of the salt treatment group, and *CB* is the biomass of the control group.

The salt tolerance coefficient (*ω*) is the ratio of the average measured value between the salt treatment group and the control group.

The membership function value of each Comprehensive index was calculated using the following formula:


(2) $$\mu \;(X_{j})=\frac{X_{j}–X_{min}}{X_{max}–\;X_{min}}$$

(3) $$\mathrm{or}\;\mu \;(X_{j})=\frac{X_{max}–X_{j}}{X_{max}–\;X_{min}}$$

where *μ (X_j_)* represents the membership function value of the comprehensive index of *j*, *X_j_* represents the comprehensive index value of *j*, and *X_min_* indicates the minimum value of *j*. *X_max_* indicates the maximum value of *j*. The positive correlation between the indexes and salt tolerance was calculated using MFA (Formula 2). The negative correlation between the indexes and salt tolerance was calculated using MFA (Formula 3).

The weight value (*W*) of each composite indicator was calculated using the following formula:


(4) $$W_{j}=\frac{V_{j}}{\sum_{j=1}^{m}V_{j}}$$

where: *W_j_* denotes the weight of the composite indicator *j* among all composite indicators; *V_j_* denotes the contribution percentage of each part of the composite indicator *j* of the material obtained through PCA.

The salt tolerance index (*D*) was calculated using the following formula:


(5) $$D=\sum_{j=1}^{n}\mu \left(X_{j}\right)\times W_{j}$$

The membership function value of each comprehensive index (*μ*), the weight value (*W*) and the salt tolerance index (*D*) of each composite indicator were calculated with reference to the methods of Chen et al. (Chen et al., 2023).

Structural equation modeling (SEM) was employed to analyze the effects of salt stress on plant growth index, physiological index, and protective enzyme system. Before the SEM procedure, variables dimensionality reduction was conducted of by PCA. The parameters of the first principal component were then used in the SEM model. During SEM analyses, the data were fitted to the models using the maximum likelihood estimation method by comparing the model-implied variance–covariance matrix against the observed variance–covariance matrix. All SEM analyses were performed using Amos version 17.0.2 (Amos Development Corporation, Chicago, IL, USA). Model fit was assessed using the chi-square (*χ*^2^) test, comparative fit index (CFI), root square mean error of approximation (RSMEA), and goodness of fit index (GFI) (Mi et al., 2015).

## 3 Results and analysis

### 3.1 Effects of NaCl stress on morphological characteristics of two legume species seedlings

**Figure 1:**
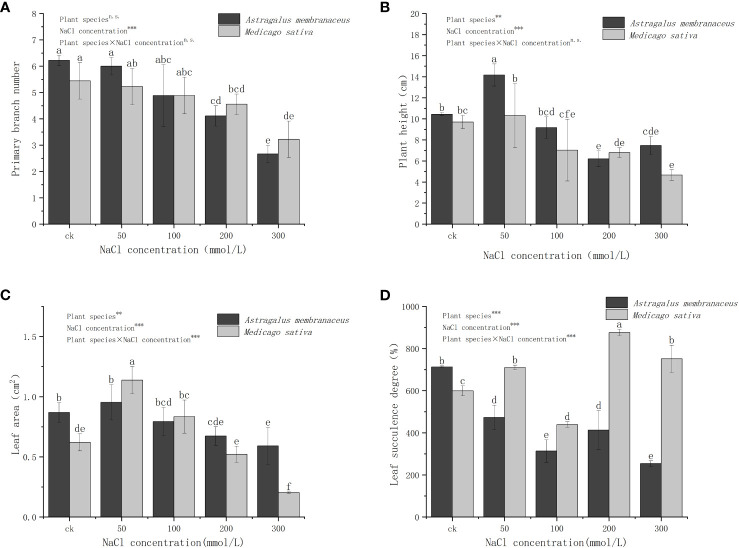
Changes in the **(A)** primary branches number, **(B)** plant height, **(C)** leaf area, and **(D)** leaf succulence degree of the two legume species under NaCl stress. Different lowercase letters indicate significant differences between treatments (*P*<0.05). Through the two-way analysis of variance, the significance of the effect of factors on the indicators is marked as ***(*P*<0.001), **(*P*<0.01), *(*P*<0.05) and n.s. (*P*>0.05), the same below.

With increasement of NaCl concentration, the PBN of both legume species gradually decreased (**Figure 1A**). The primary branch numbers of *A. membranaceus* and *M. sativa* showed no significant difference compared to the control group at NaCl concentrations of 50 mmol/L and 100 mmol/L. However, a considerable difference emerged at 200 mmol/L and 300 mmol/L NaCl concentrations (*P<*0.05). The variation in PBN among treatments was attributed to NaCl concentration (*P<*0.001). *A. membranaceus* exhibited the highest PH at 50 mmol/L and the lowest at 200 mmol/L (**Figure 1B**). The PH of *M. sativa* decreased with increasing concentration, showing no significant difference at 50 mmol/L compared to the control group. However, at NaCl concentrations of 100 mmol/L, 200 mmol/L, and 300 mmol/L, there were significant differences from the control group (*P<*0.05). The differences in PH among treatments were influenced by species (*P<*0.01) and NaCl concentration (*P<*0.001).

The LA of both plants initially increased and then decreased with rising salt concentration (**Figure 1C**), reaching its maximum at 50 mmol/L. At this concentration, the LA of *M. sativa* significantly differed from the control group (*P<*0.05). The LA of *A. membranaceus* decreased by 22.55% compared with the control group at 200 mmol/L and 300 mmol/L NaCl concentrations (*P<*0.05) and 31.99% (*P<*0.05). The LA of *M. sativa* decreased by 12.49% (*P<*0.05) and 65.96% (*P<*0.05) at 200 mmol/L and 300 mmol/L salt concentrations, respectively. The differences in LA among treatments were influenced by species (*P<*0.01), NaCl concentration (*P<*0.001), and their interactions (*P<*0.001). The LSD of *A. membranaceus* in all salt treatment groups was significantly lower than that in the control group (*P<*0.05; **Figure 1D**). Under all of salt treatments, *M. sativa's* LSD was significantly higher than that of *A. membranaceus* (*P*<0.05). At 100 mmol/L salt concentration, the LSD of *M. sativa* was the lowest and significantly lower than that of control group (*P*<0.05). At 50 mmol/L, 200 mmol/L, and 300 mmol/L salt concentrations, it was significantly higher than the control group (*P<*0.05). The differences in LSD among treatments were influenced by species (*P<*0.001), NaCl concentration (*P<*0.001), and their interactions (*P<*0.001).

### 3.2 Effects of NaCl stress on seedling biomass of the two legume species

**Figure 2:**
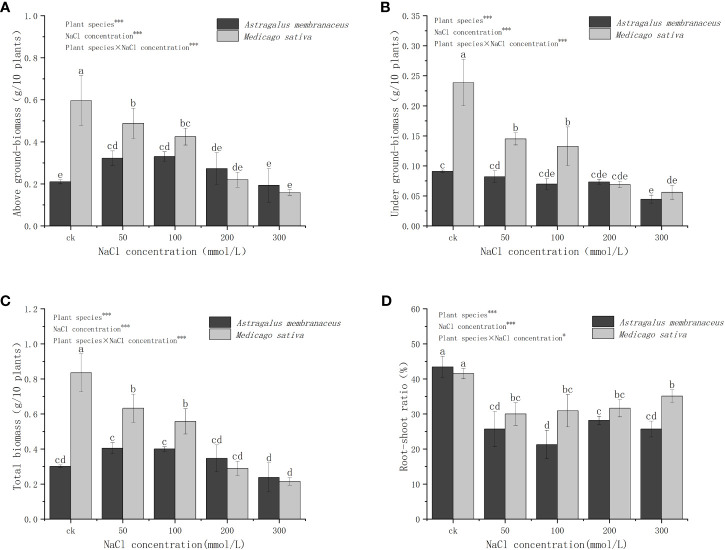
Changes in the **(A)** above ground biomass, **(B)** under ground biomass, **(C)** total biomass, and **(D)** root-shoot ratio of the two legume species under NaCl stress.

The results indicate varying trends in the overall change of different legumes under different salt concentrations (**Figure 2**). Specifically, the aboveground biomass (AGB) of *A. membranaceus* initially increased and then decreased with rising salt concentration, whereas the underground biomass (UGB) showed insignificant changes. In contrast, both AGB and UGB of *M. sativa* gradually decreased with increasing salt concentration, significantly dropping at 50 mmol/L and 100 mmol/L concentrations (*P<*0.05), and exhibiting higher levels in the 50 mmol/L and 100 mmol/L treatment groups compared to the stress of 200 mmol/L and 300 mmol/L. Total biomass and AGB trends were similar between the two legume species. The R/S of *A. membranaceus* and *M. sativa* in all NaCl-treated groups was significantly lower than in the control (*P<*0.05), with no significant differences among various salt concentration treatment groups. The variations in AGB, UGB, total biomass, and R/S among treatments were influenced by species (*P<*0.001), NaCl concentration (*P<*0.001), and their interactions (*P<*0.05).

**Figure 3:**
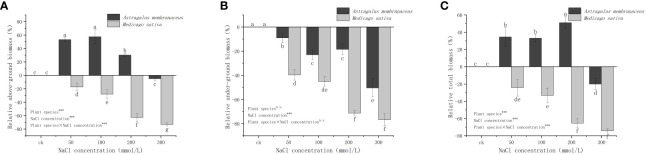
Relative changes in the **(A)** above ground biomass, **(B)** under ground biomass, and **(C)** total biomass of the two legume species under NaCl stress.

**Figure 3** illustrates the different trends in the relative biomass (Formula 1) of the two legume seedlings under NaCl stress. The relative AGB of *A. membranaceus* was significantly higher than that of the control, except at 300 mmol/L salt concentration, while the UGB was lower than that of the control group. In contrast, the aboveground and UGB of *M. sativa* was consistently lower than that of the control. The trend in total relative biomass of the two species mirrored that of AGB. *A. membranaceus* exhibited increased total biomass under 50 mmol/L, 100 mmol/L, and 200 mmol/L salt stress (34.44%, 33.00%, and 51.17% higher than the control, respectively), but under 300 mmol/L salt stress, total biomass decreased by 20.93% compared to the control. *M. sativa* showed a decrease in total biomass at 50 mmol/L, 100 mmol/L, 200 mmol/L, and 300 mmol/L NaCl concentrations (24.19%, 33.21%, 65.39%, and 74.25% lower than the control, respectively).

### 3.3 Effects of NaCl stress on physiological indexes and defense system enzymes of two legume seedlings

**Figure 4:**
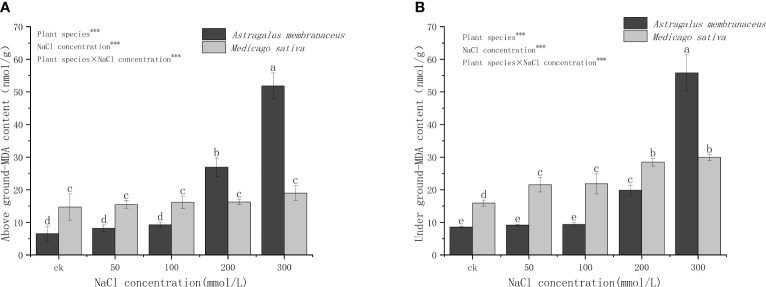
Changes in **(A)** above ground MDA content and **(B)** under ground MDA content in the two legume species under NaCl stress.

**Figure 4** reveals significant differences in the MDA content in the aboveground parts of the two legumes. Specifically, at 50 mmol/L and 100 mmol/L salt concentrations, *M. sativa* had a higher MDA content than *A. membranaceus*. At 300 mmol/L salt concentration, the MDA content of *A. membranaceus* was significantly higher than that of *M. sativa*, while the content of *A. membranaceus* did not significantly differ at 0–100 mmol/L salt concentration. There were notable differences in the content of MDA in the underground parts of the two plants. The MDA content of *A. membranaceus* showed no significant difference at 50 mmol/L and 100 mmol/L salt concentrations, but it increased exponentially at 200 mmol/L and 300 mmol/L salt concentrations. The underground MDA content of *M. sativa* was significantly higher at 200 mmol/L and 300 mmol/L salt concentrations than at 50 mmol/L and 100 mmol/L salt concentrations, and the trend of plant MDA content was similar above and below ground.

**Figure 5:**
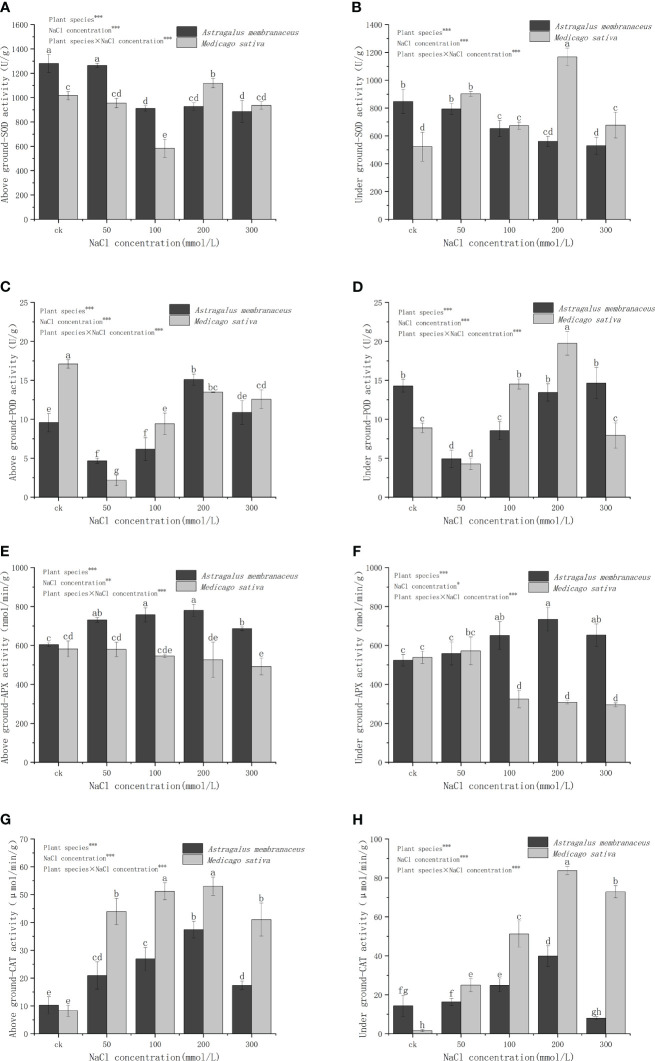
Changes in **(A)** above ground SOD activity, **(B)** under ground SOD activity, **(C)** above ground POD activity, **(D)** under ground POD activity, **(E)** above ground APX activity, **(F)** under ground APX activity, **(G)** above ground CAT activity, and **(H)** under ground CAT activity of two legume species under NaCl stress.

With an elevation in NaCl concentration, the SOD activity in the aboveground organs of *A. membranaceus* gradually diminishes (**Figure 5A**). At a concentration of 50 mmol/L, the SOD activity in the aboveground parts of plants exhibits no significant deviation from that in the control group and is notably higher than that in other salt treatment groups. *M. sativa* records the lowest SOD activity at a concentration of 100 mmol/L. Under diverse salt concentrations of *A. membranaceus*, SOD activity in the underground parts of plants progressively diminishes with the escalating salt concentration (**Figure 5B**), displaying no significant distinctions at 100 mmol/L, 200 mmol/L, and 300 mmol/L salt concentrations (*P>*0.05). The SOD activity of *M. sativa* roots in the treatment group surpasses that in the control group, reaching its peak at a concentration of 200 mmol/L.

POD activity in the aboveground part of *A. membranaceus* systematically rises with the escalating salt concentration, reaching its zenith at 200 mmol/L (**Figure 5C**). The POD activity of *M. sativa* stems and leaves gradually increases but remains significantly lower than that of the control group (*P<*0.05). POD activity of *A. membranaceus* roots is significantly lower than that of the control group at concentrations of 50 mmol/L and 100 mmol/L (*P<*0.05; **Figure 5D**). The POD activity of *M. sativa* roots is notably lower than that of the control group at a concentration of 50 mmol/L (*P<*0.05). The POD activity of *M. sativa* roots at 100 mmol/L and 200 mmol/L (reaching the highest value) is significantly higher than that of the control group (*P<*0.05).

The aboveground APX activities in all salt-treated groups of *A. membranaceus* are significantly higher than those in the control group (*P<*0.05; **Figure 5E**). The APX activity of stems and leaves of *M. sativa* is notably lower than that of the control group only at a 300 mmol/L salt concentration (*P<*0.05). The root APX activity of *A. membranaceus* is significantly higher than that of the control group at concentrations of 100 mmol/L, 200 mmol/L, and 300 mmol/L (*P<*0.05; **Figure 5F**). The root APX activity of *M. sativa* at concentrations of 100 mmol/L, 200 mmol/L, and 300 mmol/L is significantly lower than that of the control group (*P<*0.05).

The CAT activity of stems and leaves of *A. membranaceus* under all salt treatments was significantly higher than that of the control group (*P<*0.05; **Figure 5G**). The CAT activity reached its peak value at a 200 mmol/L salt concentration. Similarly, the CAT activity of the stems and leaves of *M. sativa* treated with all salt concentrations was significantly higher than that of the control group (*P<*0.05), with the highest values observed at 200 mmol/L concentration. Generally, after salt treatment, the CAT activity of *M. sativa* stems and leaves was significantly higher than that of *A. membranaceus*. The CAT activity of *A. membranaceus* root was significantly higher than that of the control group at 100 mmol/L and 200 mmol/L salt concentrations (*P<*0.05; **Figure 5H**). The maximum value was reached at the 200 mmol/L salt concentration. The root CAT activity of *M. sativa* under all salt concentrations was also significantly higher than that of the control group (*P<*0.05), reaching its peak at 200 mmol/L salt concentration.

### 3.4 Correlation analysis among parameters

Correlation analyses were conducted on seedling stage indexes of *A. membranaceus* (**Supplementary Figure 1A**), revealing significant associations. The PBN exhibited a noteworthy correlation with UGB, plant SOD activity, and LSD (*P<*0.05). Additionally, UGB showed a significant correlation with the MDA content of plants (*P<*0.05). LA demonstrated significant correlations with SOD activity in roots, MDA content in stems and leaves, and PH (*P<*0.05). The R/S was also found to be significantly correlated with LSD (*P<*0.05). Furthermore, plant SOD activity exhibited a significant correlation with root APX activity (*P<*0.05), and plant CAT activity was significantly correlated with stems and leaves APX activity (*P<*0.05).

In the case of *M. sativa* seedling stage (**Supplementary Figure 1B**), each index was subjected to correlation analysis. The PBN was significantly correlated with PH, biomass, MDA content, and APX activity stems and leaves (*P<*0.05). Biomass displayed significant correlations with plant APX activity, plant CAT activity, and plant MDA content (*P<*0.05). PH exhibited significant correlations with AGB, MDA content in stems and leaves, and APX activity (*P<*0.05). The R/S showed a significant correlation with CAT activity of the stems and leaves (*P<*0.05). Moreover, the MDA content, APX activity, and CAT activity of roots were significantly correlated with each other (*P<*0.05).

### 3.5 Principal component analysis and membership function analysis of indices of two legume plants under salt stress

The PCA load chart depicts the correlation coefficients between the original variables and the principal components. For *A. membranaceus* seedlings, the first three eigenvalues were 47.38%, 26.31%, and 13.78%, respectively, resulting in a cumulative contribution rate of 87.47% (**Supplementary Table 5**, **Supplementary Figure 2**). The eigenvector of the load diagram of the PCA and the contribution rate of each principal component revealed that PBN, SOD activity in plant stems, leaves, and roots exhibited higher loads on the first principal component. The second principal component was characterized by APX activity in stems and leaves, AGB, total biomass. The third principal component was associated with CAT activity in roots, POD activity in stems and leaves, and R/S had higher loads (**Supplementary Table 6**).

According to the load diagram of PCA of *M. sativa* seedlings, the first four eigenvalues were 46.54%, 17.43%, 13.24%, and 8.80%, leading to a cumulative contribution rate was 85.63% (**Supplementary Table 5**, **Supplementary Figures 3A, B**). Therefore, the four principal components were selected as comprehensive indexes to evaluate *M. sativa*. According to the eigenvector of the load diagram of PCA and the contribution rate of each principal component, the load diagram indicates that root CAT activity, total biomass, UGB, had higher loads on the first principal component. POD activity in stems and leaves, R/S and LA contributed more to the second principal component. SOD activity of stems and leaves, LSD, and PH exhibited higher loads in the third principal component. POD activity in plant stems, leaves and roots and MDA activity of stems and leaves contributed more to the fourth principal component. Extracting these four principal components could effectively represent the information from all indicators, allowing the use of four new variables to replace the original eighteen variables (**Supplementary Table 6**). However, each load vector represents only the correlation coefficient between the principal component and the corresponding variable, not the coefficient corresponding to each index in each principal component.

The highest salt tolerance index (*D*) (Formula 5) values were observed at control for both legume plants, indicating that salt treatment reduced the salt tolerance of the plants (**Table 1**). In the four salt treatment groups, the highest *D* values of *A. membranaceus* and *M. sativa* occurred at concentrations of 50 mmol/L and 200 mmol/L, respectively. The salt tolerance of *M. sativa* was higher than that of *A. membranaceus* under each concentration treatment in the comprehensive evaluation.

**Table 1:** Comprehensive index value, membership function value and salt tolerance evaluation value of seedling stage under different salt concentration treatments.

	Comprehensive index value				Membership function value				*D*
	x1	x2	x3	x4	μ1	μ2	μ3	μ4	
AM CK	-0.178	-0.512	1.964	0.597	0.402	0.140	1.000	0.724	0.486
AM 50	-0.555	-0.379	1.124	-1.296	0.283	0.185	0.735	0.051	0.307
AM 100	-0.626	-0.261	-0.403	-1.178	0.261	0.223	0.254	0.093	0.225
AM 200	-0.768	-0.403	-0.557	0.248	0.216	0.176	0.205	0.600	0.258
AM 300	-1.453	-0.936	-1.209	0.647	0.000	0.000	0.000	0.742	0.107
MS CK	-0.637	2.085	0.515	1.373	0.257	1.000	0.543	1.000	0.629
MS 50	0.555	0.924	-0.018	-1.439	0.633	0.616	0.375	0.000	0.488
MS 100	0.889	1.028	-1.149	-0.318	0.739	0.650	0.019	0.399	0.529
MS 200	1.717	-0.909	0.270	0.450	1.000	0.009	0.466	0.672	0.570
MS 300	1.055	-0.636	-0.536	0.914	0.791	0.099	0.212	0.837	0.492

AM mean *A. membranaceus* and MS mean *M. sativa*. D mean the salt tolerance index.

### 3.6 Structural equation modeling analysis of pathways of the two legume plants under salt stress

**Figure 6:**
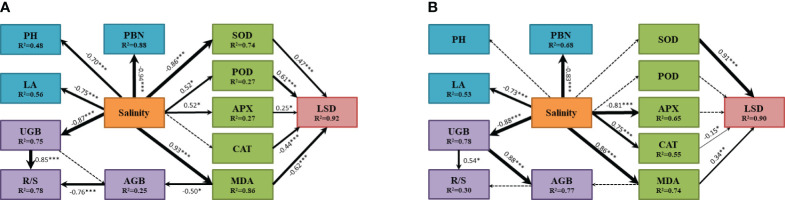
The influence pathway of plant morphological characteristics, biomass, antioxidant enzyme system and MDA of two legume species under salt stress fitted by SEM analysis (A for *Astragalus membranaceus* and B for *Medicago sativa*). The results of model fitting were (**A**: χ^2 =^ 206.859, df=62, *P*=0.242; **B**: χ^2 =^ 181.459, df=62, *P*=0.138). The arrows represent the action path relationship between the factors. The thickness of the solid arrow represents the standardized path coefficient, and the significance is marked as ***(*P<*0.001), **(*P<*0.01) and *(*P<*0.05). The dashed lines represent insignificant hypothetical regression relationships between the factors. R^2^ values indicate the proportion of variation explained by the relationships with other variables. Values associated with solid arrows represent standardized paths coefficients. PH mean plant height, LA mean leaf area, PBN mean number of primary branches, AGB mean above-ground biomass, UGB mean under-ground biomass, R/S mean root-shoot ratio, LSD mean degree of leaf succulence, SOD mean superoxide dismutase, POD mean peroxidase, APX mean ascorbate peroxidase, CAT mean Catalase, MDA mean malondialdehyde.

SEM of *A. membranaceus* indicated a high fit (χ^2^ = 206.859, df=62, *P*=0.242; **Figure 6A**). The results showed that salinity significantly negatively affected PBN, PH, and LA, explaining 88%, 48%, and 56% of their variances, respectively. Salinity had a significant positive effect on MDA, POD, and APX and adversely affected SOD. SOD, POD, APX, CAT, and MDA significantly influenced LSD, with MDA and CAT negatively affecting LSD and SOD, POD, and APX positively affecting LSD. Finally, salinity indirectly affected AGB through MDA, where MDA had an adverse effect on AGB, and Salinity directly negatively affected UGB. Both AGB and UGB significantly influenced R/S, with the negative effect of AGB and the positive effect of UGB together explaining 78% of the total variance of R/S.

SEM of *M. sativa* also demonstrated a high fit (χ^2^ = 181.459, df=62, *P*=0.138; **Figure 6B**). Salinity adversely affected PBN and LA, explaining 68% and 53% of their variances, respectively. Salinity directly affected MDA, APX, and CAT, with significant positive effects on MDA and CAT and an adverse effect on APX. Additionally, salinity significantly influenced LSD only through SOD, CAT, and MDA, which together explained 90% of the total variance of LSD. Salinity directly and adversely affected UGB, explaining 78% of its variance. Salinity indirectly affected AGB through UGB, where UGB positively affected AGB, explaining 77% of its variance. In this model, only UGB had a positive effect on R/S.

## 4 Discussion

### 4.1 Effects of salt stress on morphological characteristics of seedlings

The tolerance of plants to saline environments is often evident in their growth characteristics (Munns and Tester, 2008; Verma et al., 2023). Under adverse conditions, plants adapt through changes in morphological characteristics and growth state (Miryeganeh, 2021). In our study, morphological traits such as PBN, PH, and LA exhibited a downward trend to varying degrees under salt stress (**Figure 1**). High level of salt stress had significant and negative effect on plant growth. Plants readjust their resource allocation patterns to cope with stress under high salinity conditions (Zhang et al., 2020). Leaf fleshy refers to the enlargement of parenchyma tissue in plant organs, leading to the dilution of cell fluid, aiding plants in dealing with salt stress (Ottow et al., 2005). Results indicated that under different salt stress treatments, the LSD varied significantly between the two plants, suggesting that *M. sativa* had better water absorption and storage capacity than *A. membranaceus* under salt stress (**Figure 1**), ensuring water demand for average plant growth (Ogburn and Edwards, 2010).

### 4.2 Effects of salt stress on seedling biomass

Plants under salt stress maintain growth by adjusting biomass energy distribution, primarily by reducing carbon assimilation and altering osmotic energy consumption (Zhang et al., 2020). Generally, in saline-alkali environments, plant individual development is shortened, growth is slowed or stopped, and biomass accumulation is reduced. However, for some salt-tolerant plants, low-concentration salt stress can promote growth (Ogburn and Edwards, 2010). Our study indicated that both legumes were affected by salt stress to varying degrees, with the AGB of *A. membranaceus* slightly increasing under low salt concentrations (**Figure 2**). Slight salt stress could sometimes stimulate plant growth (Yu et al., 2020). Conversely, *M. sativa* exhibited a clear downward trend with increasing salt concentration (**Figure 2**). With increasing stress factors, plants tend to preserve the biomass of the underground part, reflected in the increase in the R/S (Rabhi et al., 2010). The underground parts of plants bear the brunt of environmental stress caused by salt, prompting many plants to adjust root morphology in response to salt stress (Zhang et al., 2023). Some plants inhibit the growth of the underground part in salt environments to avoid excessive exposure to high salt (Zhao et al., 2020). In our experiment, the biomass of the underground portion relative to the aboveground part increased at a salt concentration of 300 mmol/L. More allocation of root biomass and an increase in the R/S may have a greater potential for plant uptake of soil water under salinity stress conditions. (**Figures 2**, **3**).

### 4.3 Effects of salt stress on plasma membrane peroxidation and protective enzyme activities of seedlings

When the salt content in the plant growing environment increases, it induces changes in the cell membrane function due to salt damage. This results in an elevated rate of electrolyte exosmosis in the cell, leading to a corresponding increase in relative conductivity (Khatri and Rathore, 2022). The imbalance in free radical metabolism in the body causes an increase in the content of certain free radicals (Heyno et al., 2011; Hasanuzzaman et al., 2021). Specifically, superoxide free radicals can trigger lipid peroxidation of unsaturated fatty acids in membrane lipids, causing serious damage to the biofilm system. MDA, a membrane lipid peroxide produced in this process, serves as an indicator reflecting the strength of plant resistance under stress conditions (de Azevedo Neto et al., 2006). Various plants exhibit different levels of antioxidant enzyme activity in relation to their salt stress tolerance (Costa et al., 2010). Our study demonstrated significant differences in MDA content in *A. membranaceus*, showing an increasing trend with higher salt concentrations (**Figure 4**). Generally, under stress conditions such as salt, heavy metals, mechanical damage, and high temperature, MDA content in plants tends to increase (Tang et al., 2015). Notably, there was no significant difference in *M. sativa* for MDA content, possibly due to its high salt tolerance, which mitigates MDA changes through osmotic regulation (**Figure 4**). With increasing salt concentration, SOD activity, as the first line of defense against reactive oxygen species damage, usually increases rapidly and significantly (Xu et al., 2013). In our study, CAT and POD activities exhibited similar trends to SOD but with varying degrees of enhancement, and the change in SOD activity was not significant (**Figure 5**). The activities of CAT, POD, and APX all increased to different extents, suggesting the plant’s ability to perform ion regionalization through self-regulation and the mutual influence of osmoregulatory substances, thereby alleviating high salt stress (Malakar and Chattopadhyay, 2021).

### 4.4 Comprehensive evaluation of salt stress on salt tolerance of seedlings

The extent of tissue damage in plants responding to salt stress is often reflected in apparent morphological indexes (Xiao and Zhou, 2022). NaCl inhibits plant growth, and this study identified different degrees of damaged morphological characteristics on the fifth day of salt stress treatment. PCA revealed highly significant correlations among the indicators, indicating a certain degree of overlap and crossover in the information reflected by them. A single index was insufficient to gauge salt tolerance (Munns and Tester, 2008). Through PCA, we simplified the data structure, identified which key variables should be retained or excluded to analyze the relationship between each index and salt tolerance, determined reliable salt tolerance evaluation indicators, and adjusted the one-sidedness of a single index. Higher loading values of the indicator for each principal factor indicated a stronger correlation with its principal factors (Chen et al., 2023). In our study, the results of PCA revealed that the morphological parameters (PBN, PH and LA) for *A. membranaceu* and the biomass parameters (TB, AGB and UGB) for *M. sativa* was present in the first principal component and had a large load (**Supplementary Table 6**). In general, the parameters of morphology and biomass were the most apparent changes under salt stress (Kumar et al., 2021).

SEM for two legumes illustrated the response pathway among indices under salt stress. Salinity directly influenced the PBN, PH, and LA of the main stem. Under low-concentration salt stress, the biomass of *A. membranaceus* increased, maintaining the plant’s normal physiological function through a protective enzyme system (**Figure 6**). AGB slightly increased by reducing PH, while UGB remained relatively stable. Typically, UGB is less affected by salt stress, allowing more nutrients to be allocated to AGB, providing additional energy for stress resistance (Loudari et al., 2022). However, in our study, the increase in salt concentration did not affect the PH of *M. sativa*, and the inhibiting effect of salinity on both aboveground and UGB was synchronized (**Figure 6**). Variations in stress-tolerant growth strategies among species led to different responses to apparent traits, particularly interspecific variation and environmentally controlled biomass allocation processes (Poorter et al., 2012; Tang et al., 2022). Based on the results of comprehensive salt tolerance analysis, the *D* value decreased with an increase in salt stress concentration, indicating a rise in salt stress and a decrease in plant salt tolerance (**Table 1**). Simultaneously, the *D* value of *M. sativa* exhibited a slight rebound at 100–200 mmol/L. The comprehensive evaluation of salt tolerance results suggested that under the stimulation of a specific threshold salt concentration, plants would gradually establish a tolerance mechanism through a physiological response (Sohrabi et al., 2012). However, our experimental results may have some potential limitations, including the typicality of legume species, the selected parameters, and the control of soil nutrient conditions, etc. We will continue to investigate the salt tolerance mechanism of legumes in future studies.

## 5 Conclusion

Salt stress significantly affected the PBN, PH, LA, and LSD of the two legume plants. Salt stress directly affected the energy distribution of plant roots, stems, and leaves. Under salt stress, the two legumes exhibited different response strategies in protective enzyme system and potential cell membrane damage. Two simplified evaluation index systems for the two legumes seedling growth salt tolerance were screened and identified. The primary evaluation parameters for *A. membranaceus* seedlings were PBN, APX and CAT, whereas for *M. sativa* were CAT, POD and SOD. *M. sativa* was more salt tolerant than *A. membranaceus* based on salt tolerance integration scores.

## Data availability statement

The original contributions presented in the study are included in the article/**Supplementary Material**. Further inquiries can be directed to the corresponding author.

## Author contributions

JM: Methodology, Project administration, Supervision, Writing – original draft, Writing – review & editing. XR: Data curation, Investigation, Writing – original draft. JS: Writing – review & editing. FW: Investigation, Writing – original draft. QW: Investigation, Writing – original draft. HP: Investigation, Writing – original draft. LK: Formal Analysis, Writing – review & editing. CW: Writing – review & editing.
