# Supplementary material for: An insight into the different responses to salt stress in growth characteristics of two legume species during seedling growth
Source: Front Plant Sci. 2024 Jan 24;14:1342219. doi: 10.3389/fpls.2023.1342219 (PMC10847288; doi:10.3389/fpls.2023.1342219)
Supplement: Supplementary file 1 [file DataSheet_1.docx]

Supplementary Material

**Supplementary Table 1.** Regression equation between various indexes of *Astragalus membranaceus* and salt concentration.

| Indexes | Regression equation | Slope | R^2^ | *P* |
| --- | --- | --- | --- | --- |
| PBN | Y^2^=5.885-0.010x | -0.010 | 0.741 | 0.061 |
| PH | Y^2^=11.865-0.018x | -0.018 | 0.513 | 0.173 |
| LA | Y^2^=0.923-0.001x | -0.001 | 0.868 | 0.021* |
| AGB | Y^2^=0.291-0.001x | -0.001 | 0.132 | 0.547 |
| UGB | Y^2^=0.090-0.001x | -0.001 | 0.853 | 0.025* |
| TB | Y^2^=0.380-0.001x | -0.001 | 0.308 | 0.332 |
| R/S | Y^2^=0.333-0.001x | -0.001 | 0.236 | 0.406 |
| LSD | Y^2^=5.854-0.012x | -0.012 | 0.626 | 0.111 |
| AMDA | Y^2^=0.545+0.154x | 0.154 | 0.915 | 0.011* |
| UMAD | Y^2^=0.936+0.151x | 0.151 | 0.804 | 0.039* |
| ASOD | Y^2^=1231.772-1.370x | -1.370 | 0.680 | 0.086 |
| USOD | Y^2^=820.067-1.104x | -1.104 | 0.900 | 0.014* |
| APOD | Y^2^=6.792+0.019x | 0.019 | 0.311 | 0.328 |
| UPOD | Y^2^=9.095+0.016x | 0.016 | 0.202 | 0.448 |
| AAPX | Y^2^=686.04+0.198x | 0.198 | 0.117 | 0.573 |
| UAPX | Y^2^=556.383+0.519x | 0.519 | 0.556 | 0.148 |
| ACAT | Y^2^=18.614+0.030x | 0.030 | 0.127 | 0.556 |
| UCAT | Y^2^=20.157+0.004x | 0.004 | 0.002 | 0.950 |

The regression relationship is significantly represented as *(*P*<0.05).

**Supplementary Table 2.** Regression equation between various indexes of *Medicago sativa* and salt concentration.

| Indexes | Regression equation | Slope | R^2^ | *P* |
| --- | --- | --- | --- | --- |
| PBN | Y^2^=5.572-0.008x | -0.008 | 0.946 | 0.005** |
| PH | Y^2^=10.00-0.018x | -0.018 | 0.856 | 0.024* |
| LA | Y^2^=0.945-0.002x | -0.002 | 0.558 | 0.147 |
| AGB | Y^2^=0.572-0.002x | -0.002 | 0.965 | 0.003** |
| UGB | Y^2^=0.201-0.001x | -0.001 | 0.845 | 0.027* |
| TB | Y^2^=0.773-0.002x | -0.002 | 0.946 | 0.005** |
| R/S | Y^2^=0.350-0.001x | -0.001 | 0.057 | 0.698 |
| LSD | Y^2^=5.827+0.007x | 0.007 | 0.269 | 0.371 |
| AMDA | Y^2^=14.638+0.013x | 0.013 | 0.886 | 0.017* |
| UMAD | Y^2^=17.631+0.045x | 0.045 | 0.918 | 0.010* |
| ASOD | Y^2^=896.157+0.197x | 0.197 | 0.014 | 0.851 |
| USOD | Y^2^=706.655+0.630x | 0.630 | 0.901 | 0.621 |
| APOD | Y^2^=10.045+0.007x | 0.007 | 0.022 | 0.812 |
| UPOD | Y^2^=9.286+0.014x | 0.014 | 0.074 | 0.659 |
| AAPX | Y^2^=585.220-0.309x | -0.309 | 0.964 | 0.003** |
| UAPX | Y^2^=528.244-0.927x | -0.927 | 0.676 | 0.087 |
| ACAT | Y^2^=29.257+0.079x | 0.079 | 0.272 | 0.367 |
| UCAT | Y^2^=14.327+0.250x | 0.250 | 0.791 | 0.043* |

The regression relationship is significantly represented as **(*P*<0.01) and *(*P*<0.05).


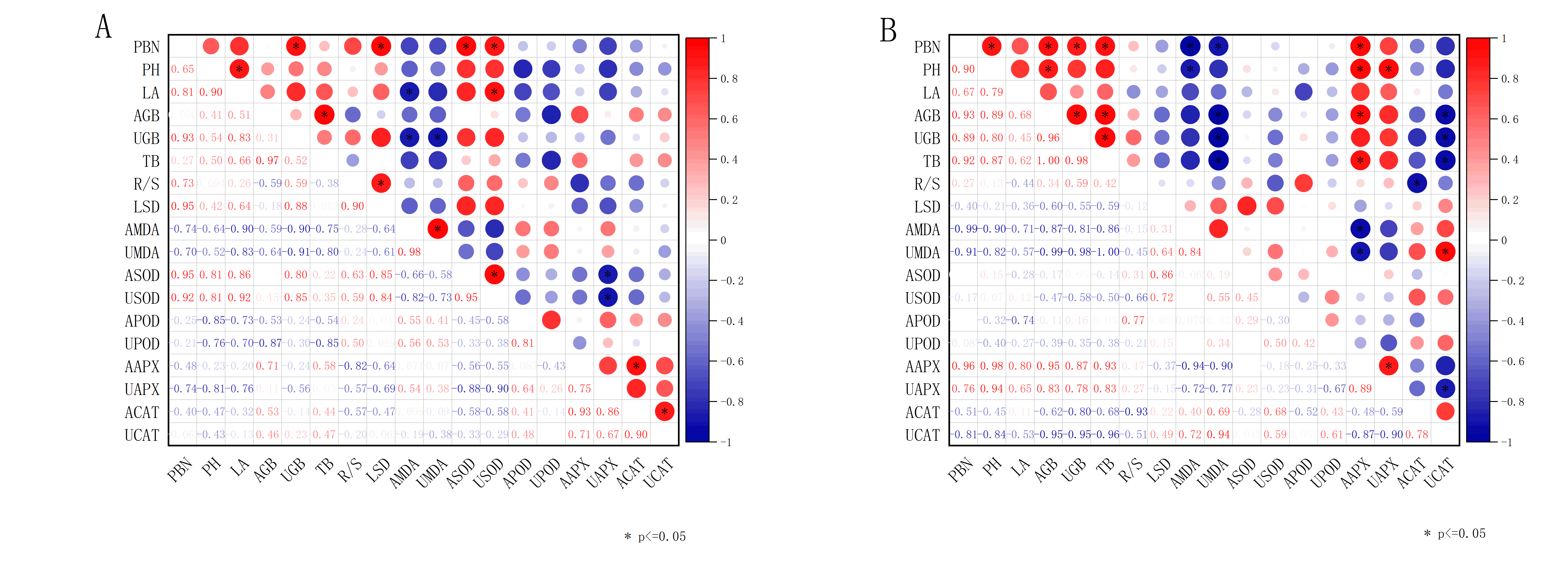


**Supplementary Figure 1.** Correlation analysis of growth indexes of two legume plants at seedling stage (A for *Astragalus membranaceus* and B for *Medicago sativa*).

**Supplementary Table 3.** Salt tolerance coefficient of *Astragalus membranaceus* seedlings under different salt concentrations.

| Indexes | Salt concentration | | | |
| --- | --- | --- | --- | --- |
|  | 50 mmol/L | 100 mmol/L | 200 mmol/L | 300 mmol/L |
| PBN | 1.00±0.06a | 0.81±0.20ab | 0.69±0.06b | 0.44±0.06c |
| PH | 1.36±0.10a | 0.88±0.10b | 0.59±0.07c | 0.72±0.08bc |
| LA | 1.10±0.17a | 0.91±0.13ab | 0.77±0.09b | 0.68±0.18b |
| AGB | 1.54±0.16a | 1.57±0.10a | 1.30±0.36ab | 0.92±0.38b |
| UGB | 0.90±0.11a | 0.77±0.10a | 0.81±0.04a | 0.49±0.07b |
| TB | 1.34±0.11a | 1.33±0.04a | 1.15±0.26ab | 0.79±0.28b |
| R/S | 0.59±0.12a | 0.49±0.09a | 0.65±0.03a | 0.59±0.05a |
| LSD | 0.66±0.08a | 0.44±0.07bc | 0.58±0.13ab | 0.36±0.02c |
| AMDA | 1.25±0.16c | 1.42±0.12c | 4.12±0.44b | 7.95±0.61a |
| UMAD | 1.07±0.04c | 1.09±0.07c | 2.31±0.19b | 6.51±0.65a |
| ASOD | 0.99±0.01a | 0.71±0.02b | 0.72±0.02b | 0.69±0.07b |
| USOD | 0.94±0.05a | 0.77±0.07b | 0.66±0.04bc | 0.62±0.07c |
| APOD | 0.49±0.03c | 0.64±0.15c | 1.58±0.07a | 1.14±0.16b |
| UPOD | 0.34±0.08c | 0.60±0.08b | 0.94±0.08a | 1.03±0.14a |
| AAPX | 1.21±0.02ab | 1.25±0.06a | 1.29±0.05a | 1.14±0.02b |
| UAPX | 1.07±0.11b | 1.24±0.14ab | 1.40±0.12a | 1.25±0.11ab |
| ACAT | 2.05±0.48bc | 2.63±0.40b | 3.66±0.29a | 1.70±0.16c |
| UCAT | 1.14±0.13c | 1.73±0.23b | 2.78±0.37a | 0.56±0.06d |

Different lowercase letters indicate significant differences between salt treatments (*P*<0.05).

**Supplementary Table 4.** Salt tolerance coefficient of *Medicago sativa* seedlings under different salt concentrations.

| Indexes | Salt concentration | | | |
| --- | --- | --- | --- | --- |
|  | 50 mmol/L | 100 mmol/L | 200 mmol/L | 300 mmol/L |
| PBN | 0.92±0.12a | 0.86±0.12a | 0.80±0.07a | 0.57±0.12b |
| PH | 1.06±0.31a | 0.72±0.30a | 0.70±0.05a | 0.48±0.05a |
| LA | 1.83±0.18a | 1.34±0.22b | 0.84±0.11c | 0.33±0.01d |
| AGB | 0.82±0.12a | 0.71±0.07a | 0.37±0.06b | 0.32±0.02b |
| UGB | 0.61±0.04a | 0.56±0.13a | 0.29±0.02b | 0.23±0.05b |
| TB | 0.76±0.10a | 0.67±0.09a | 0.35±0.05b | 0.29±0.03b |
| R/S | 0.72±0.08a | 0.75±0.11a | 0.76±0.06a | 0.85±0.04a |
| LSD | 1.19±0.02b | 0.73±0.02c | 1.46±0.03a | 1.25±0.11b |
| AMDA | 1.05±0.08b | 1.10±0.13ab | 1.11±0.05ab | 1.29±0.16a |
| UMAD | 1.35±0.14b | 1.37±0.19b | 1.79±0.07a | 1.88±0.06a |
| ASOD | 0.94±0.04b | 0.58±0.07c | 1.10±0.04a | 0.92±0.03b |
| USOD | 1.72±0.04c | 1.29±0.05b | 2.23±0.12a | 1.30±0.18c |
| APOD | 0.13±0.04c | 0.55±0.08b | 0.79±0.03a | 0.73±0.07a |
| UPOD | 0.48±0.08d | 0.55±0.09b | 2.22±0.17a | 0.89±0.18c |
| AAPX | 1.02±0.06a | 0.55±0.10a | 0.91±0.16a | 0.85±0.07a |
| UAPX | 1.06±0.13a | 0.55±0.11b | 0.57±0.02b | 0.55±0.02b |
| ACAT | 5.33±0.57bc | 0.55±0.12ab | 6.44±0.40a | 4.98±0.72c |
| UCAT | 16.03±2.15d | 0.55±0.13c | 53.85±1.38a | 46.81±0.03b |

Different lowercase letters indicate significant differences between salt treatments (*P*<0.05).

**Supplementary Table 5.** Eigenvalues, contribution ratio and cumulative contribution ratio at seedling stage of *Astragalus membranaceus* and *Medicago sativa* obtained by principal component analysis.

| Species | Principal factors | Eigenvalue | Contribution ratio (%) | Cumulative contribution ratio (%) |
| --- | --- | --- | --- | --- |
| *Astragalus membranaceus* | 1 | 8.527 | 47.375 | 47.375 |
|  | 2 | 4.736 | 26.314 | 73.688 |
|  | 3 | 2.481 | 13.781 | 87.469 |
| *Medicago*  *sativa* | 1 | 8.378 | 46.542 | 46.542 |
|  | 2 | 3.137 | 17.425 | 63.967 |
|  | 3 | 2.383 | 13.241 | 77.209 |
|  | 4 | 1.516 | 8.423 | 85.632 |

**Supplementary Table 6.** Eigenvector at seedling stage of *Astragalus membranaceus* and *Medicago sativa* based on principal component analysis.

| Items | *Astragalus membranaceus* | | |  | *Medicago sativa* | | | |
| --- | --- | --- | --- | --- | --- | --- | --- | --- |
| Principal factors | 1 | 2 | 3 |  | 1 | 2 | 3 | 4 |
| PBN | 0.312 | 0.053 | 0.104 |  | 0.262 | 0.118 | 0.207 | 0.308 |
| PH | 0.291 | 0.039 | -0.279 |  | 0.094 | 0.117 | -0.366 | 0.099 |
| LA | 0.265 | 0.014 | 0.013 |  | 0.197 | 0.436 | 0.119 | 0.012 |
| AGB | 0.103 | 0.394 | -0.094 |  | 0.325 | 0.057 | -0.012 | 0.015 |
| UGB | 0.288 | 0.022 | 0.288 |  | 0.328 | -0.100 | -0.016 | 0.078 |
| TB | 0.159 | 0.370 | -0.024 |  | 0.335 | 0.012 | -0.014 | 0.034 |
| R/S | 0.152 | -0.325 | 0.294 |  | 0.138 | -0.477 | -0.043 | 0.027 |
| LSD | 0.252 | -0.176 | 0.291 |  | -0.203 | -0.105 | 0.470 | -0.118 |
| AMDA | -0.301 | -0.142 | -0.152 |  | -0.162 | -0.069 | -0.290 | -0.402 |
| UMAD | -0.274 | -0.191 | -0.244 |  | -0.327 | 0.033 | 0.032 | -0.145 |
| ASOD | 0.306 | -0.111 | 0.008 |  | -0.055 | -0.249 | 0.551 | -0.062 |
| USOD | 0.311 | -0.066 | 0.020 |  | -0.195 | 0.224 | 0.347 | 0.184 |
| APOD | -0.222 | -0.107 | 0.417 |  | -0.015 | -0.491 | -0.073 | 0.314 |
| UPOD | -0.183 | -0.312 | 0.280 |  | -0.167 | -0.012 | -0.030 | 0.675 |
| AAPX | -0.123 | 0.404 | 0.034 |  | 0.213 | 0.152 | 0.101 | 0.065 |
| UAPX | -0.247 | 0.179 | 0.177 |  | 0.281 | 0.062 | 0.221 | -0.266 |
| ACAT | -0.155 | 0.337 | 0.253 |  | -0.241 | 0.377 | -0.078 | 0.102 |
| UCAT | -0.070 | 0.274 | 0.464 |  | -0.336 | 0.035 | -0.073 | 0.117 |


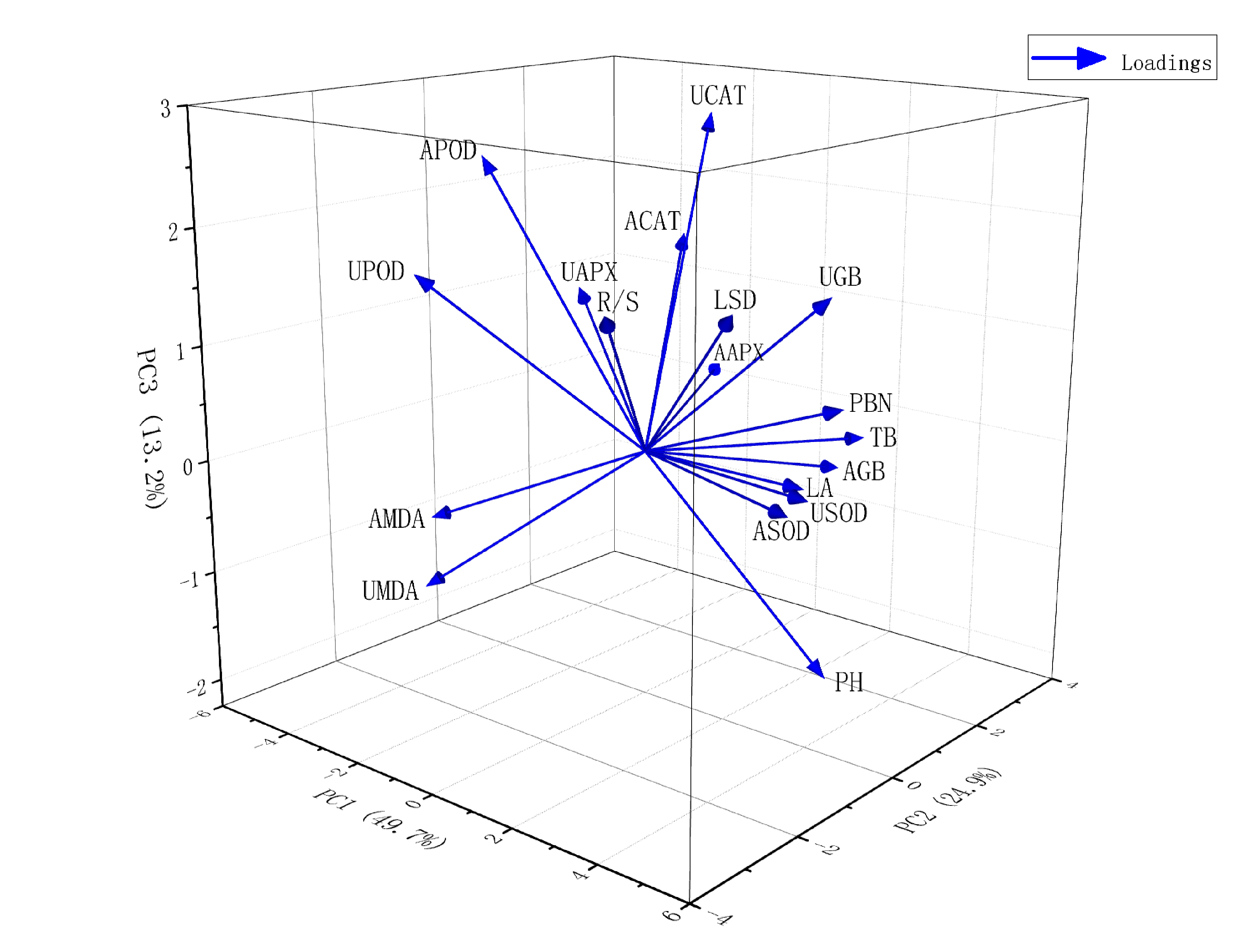


**Supplementary Figure 2.** Indexes loading results of principal component analysis of *Astragalus membranaceus* seedlings.


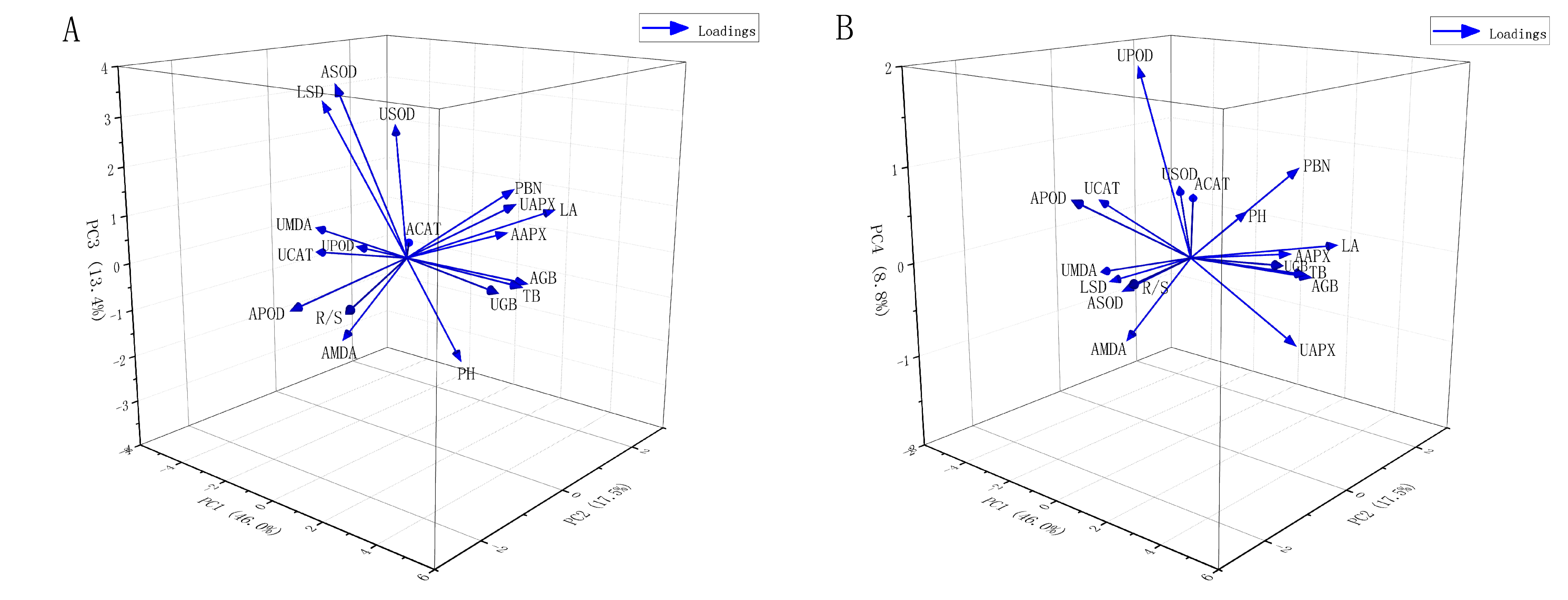


**Supplementary Figure 3.** Indexes loading results of principal component analysis of *Medicago sativa* seedlings.


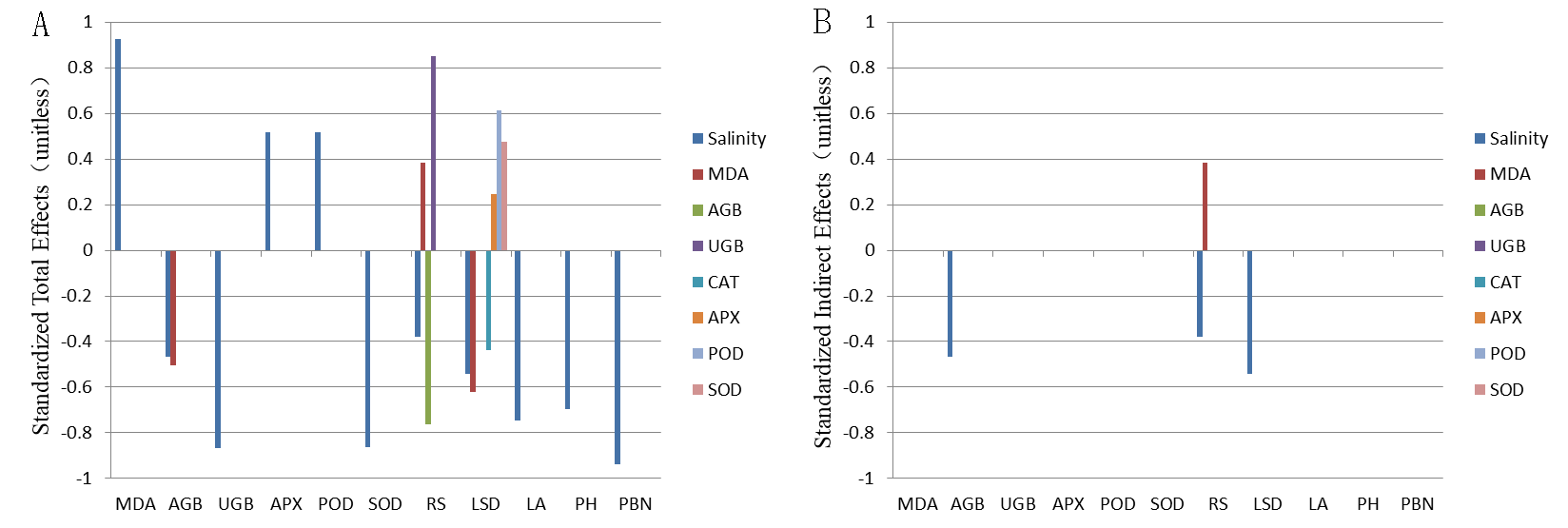


**Supplementary Figure 4.** The total and indirect effects of morphological characteristics, biomass and physiological indices of *Astragalus membranaceus* under salt stress based on structural equation model.


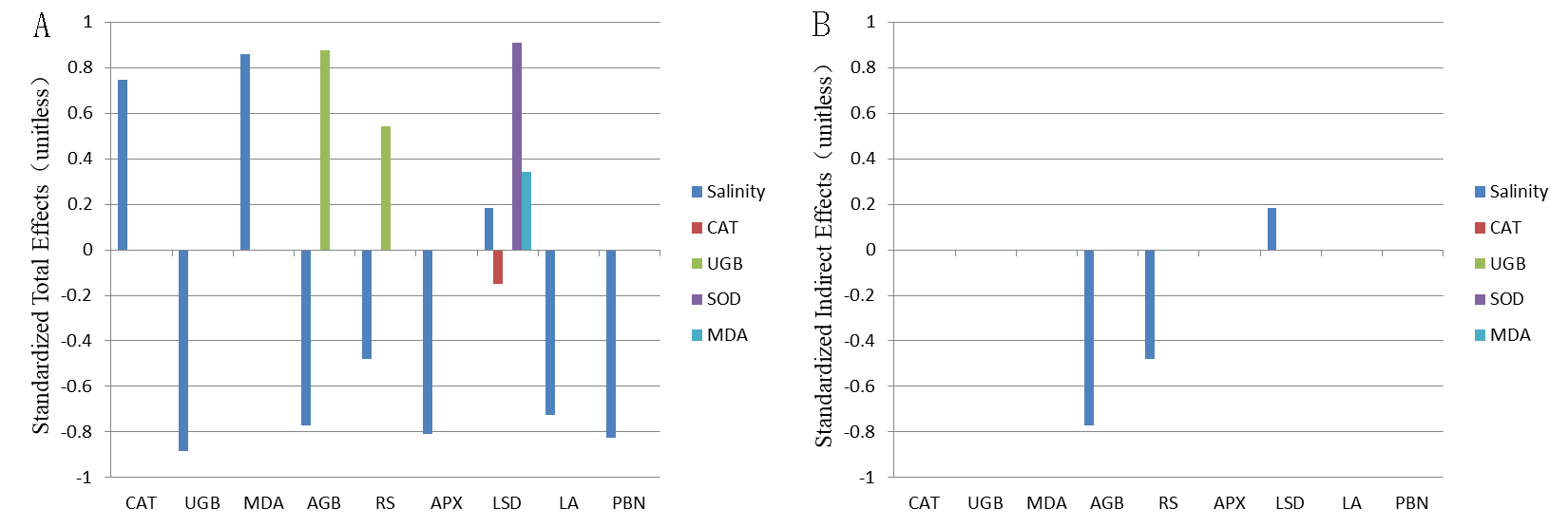


**Supplementary Figure 5.** The total and indirect effects of morphological characteristics, biomass and physiological indices of *Medicago sativa* under salt stress based on structural equation model.
